# Promoting anti-racism in the legal system: an application of the STYLE framework

**DOI:** 10.3389/fpsyg.2023.1061637

**Published:** 2023-08-29

**Authors:** Rebecca L. Fix, Idia B. Thurston, Renee M. Johnson, Stanley Andrisse

**Affiliations:** ^1^Bloomberg School of Public Health, Johns Hopkins University, Baltimore, MD, United States; ^2^Texas A&M University, College Station, TX, United States; ^3^Howard University, Washington, DC, United States; ^4^From Prison Cells to PhD, Baltimore, MD, United States

**Keywords:** equity, training, professional development, intersectionality, self-evaluation

## Abstract

Racism is a critical social problem, and we present a framework to guide professionals in engaging in anti-racist practices. Professionals on the frontlines in psychology and related fields such as social work and public health have a responsibility to engage in anti-racist practices. Part of the professional role must be to advocate for justice through increased proximity to the issues and engagement in anti-oppressive practices. The current discourse introduces a framework through which people working in psychology and other related professions can promote anti-racism work, highlighting the legal system for illustrative purposes. While some professionals in psychology may not have direct experience with the legal system, many of the individuals served by psychologists do (e.g., clients/patients, students, community members). Our framework is represented by the acronym STYLE (Self-examination, Talk about racism, Yield time to anti-racism work, Learn about structural racism, Evaluate policies and practices). The goal of STYLE is to expand anti-racism science and practice within psychology and related fields. We describe new roles for professionals in dismantling health inequities and offer specific pathways to develop critical partnerships toward this aim. STYLE explicitly encourages active, intentional involvement of affected community members in the development and evaluation of approaches to health services. To achieve equity and to promote individual and organizational growth in anti-racism and ultimately anti-oppression work, professionals must focus on changing their STYLE.

## Introduction

Racism is a public health and social problem that is deeply integrated across social systems – including the juvenile and criminal legal systems (hereafter referred to as the criminal legal system) and the broader legal system (e.g., family courts). Racism shapes the provision of services in psychology, social work, education, and public health (Ford and Airhihenbuwa, 2010; Jee-Lyn García and Sharif, 2015; American Public Health Association, 2020; Zhang et al., 2020; Olivares, 2022). Racism is also a key driver of health and social inequities or the avoidable or remediable health differences among groups of people based on their social, economic, demographic, or geographic status (American Psychological Association, 2021a,b,c; World Health Organization, 2021). Given the significance of racism as a critical public health problem (Jee-Lyn García and Sharif, 2015), those in psychology, community leadership roles, and other health professions must be at the forefront of efforts to dismantle racism. In the current article, we present STYLE – a framework for anti-racism science and practice among psychology and health professionals. We review the developmental process of STYLE, and highlight its utility in the legal system. We also provide examples of how to incorporate STYLE and plans for empirical evaluation of the STYLE framework. Finally, we emphasize the need to look beyond the policing stage of the legal system, such as in court and during incarceration, to ensure necessary broader systemic reform.

According to the American Psychological Association (APA), racism is defined as a form of prejudice that assumes members of socially-constructed racial categories have distinctive characteristics and that these chracteristics result in some racial groups being inferior to others” (American Psychological Association, 2023). Structural racism highlights that racism is embedded into social institutions; it is defined as a “...system in which public policies, institutional practices, cultural representations, and other norms work in various, often reinforcing ways to perpetuate racial group inequity” (Aspen Institute, 2004, p. 11). Structural racism provides the infrastructure for oppression and inequity, significantly harming the mental and physical health of many people in the U.S. (Barnes et al., 2008; Bailey et al., 2017). People racialized as Black, Indigenous, Latine, Asian, Native Hawaiian and Pacific Islander, and those residing in systemically oppressed communities are more likely to experience adverse health and social outcomes, including chronic and infectious disease, injury, violence, and limited educational attainment due to structural racism (Bailey et al., 2017). White professionals are also impacted by structural racism, as inculcation to racist systems limits their effectiveness in service provision and increases the likelihood that their work preserves existing patterns of oppression; it can also negatively impact White professionals’ health and well-being (Leitner et al., 2016).

The history of structural racism is long and multifaceted, spanning all of U.S. history and many centuries before (Bailey et al., 2017; Kendi and Blain, 2021). Racial inequity was recognized as significant by the United Nations, who convened the International Convention on the Elimination of All Forms of Racial Discrimination in the 1960s (Schwelb, 1966). Racism is embedded into social institutions, and the legal system reflects a fertile ground for targeting efforts to interrupt racist policies and practices that maintain structural racism (e.g., Yim et al., 2022). Professional societies including the APA, the American Public Health Association, the American Medical Association, the National Association of Social Workers, the Society for Epidemiologic Research, and others have released statements addressing racism. Those statements emphasize the need to (1) take action and to promote anti-racism practices, (2) reduce discrimination in their organizations and beyond, and (3) prevent the harmful effects of microaggressions and hate crimes in the legal system. Task forces, evidence-based resources for increased awareness and education, changes to society operations, and re-examination of training approaches and curricula through professional ethical mandates have been described as organizational priorities (e.g., American Psychological Association, 2021a,b,c; Zhang et al., 2020).

Further, this is not psychology’s first (or second, or third) reckoning with racism. Most recently, the APA adopted three critical resolutions in 2021 that support the urgency of addressing the role of racism in science, practice, education, and advocacy efforts (American Psychological Association, 2021a,b,c). Specifically, APA issued an apology to people of color for their role in promoting, perpetuating, and failing to challenge racism, racial discrimination, and human hierarchy in the U.S. (American Psychological Association, 2021b). APA also announced their commitment to dismantling systemic racism (American Psychological Association, 2021c) and advancing equity in psychology (American Psychological Association, 2021a).

## Purpose

Given that racism impacts health and social problems, professionals working in these areas need to: [1] understand how racism operates at structural and interpersonal levels and their role in maintaining systems of oppression, [2] commit to systemic and policy reform to promote racial equity, and [3] develop skills to integrate anti-racist strategies into their work, including skills pertaining to advocacy and policy reform. In this article, we introduce the “STYLE” framework to support professionals in enhancing their knowledge about racism and engaging in anti-racism work. Though the STYLE framework was formulated to be applicable across contexts, we apply the it to anti-racist approaches in the legal system and provide a template for professionals to track their growth over time.

### STYLE: development and conceptualization of a framework for anti-racism science and practice

**Figure 1:**
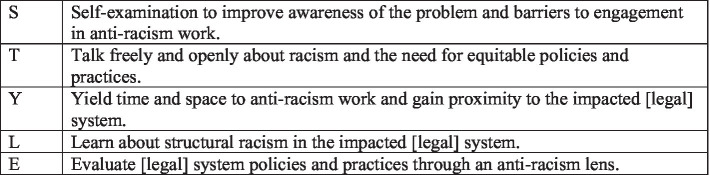
Steps toward anti-racist practice in psychology and related professions: the STYLE Framework.

There is a longstanding need for anti-racist reform across a variety of systems in the U.S., and especially the legal system. In response to the urgent need for action, we introduce STYLE, a theory- and research-driven framework for professionals to address racism stemming from and perpetuated by the legal system. The framework aims to promote development and refinement of existing structures, policies, practices, and resources to achieve a culture of anti-racism. STYLE is an acronym that stands for: Self-examination, Talk freely and openly, Yield time and space, Learn about racism, and Evaluate policies and practices (see Figure 1). STYLE offers a framework for professionals to integrate anti-racist practices and policies directly into their work.

STYLE was developed collaboratively among people who have lived experience in the legal system, professionals working within the system, and system reform advocates, and it expands on previous work by Fix et al. (2022). Our team includes people who are survivors of violent crime, who have direct experience in the legal system, and who have served people in and impacted by the legal system (these categories are not exclusive). The STYLE framework was informed by our direct experiences and the experiences and insights of individuals with lived experience.

Altogether, STYLE was borne from the need for psychologists to address racism more directly in their work (Fix et al., 2022), and has since been updated to include strategies for anyone who works in the psychological or public health fields to adopt. The first iteration of STYLE came from a group of psychologists interested in embedding anti-racist practices into their work. The first author developed the initial acronym to help psychologists think about racism in policing and how it impacts children and adolescents in pediatric clinical settings (see Fix et al., 2022). The first author then developed a set of associated skills to encompass the assigned framework areas, and the second author helped refine the definitions of each STYLE component. Then, additional partners interested in addressing anti-racism at a structural level were brought into the conversation, including coauthors and other colleagues who could help emphasize lived experiences. Through ongoing meetings, our team continues to work on testing the recommended process, and evaluating (and refining) the STYLE framework by recruiting a representative sample of people working in psychology and related fields (e.g., within distinct roles, contexts, demographic characteristics and social identifiers).

#### Author background

Adopting Williams et al. (2023) decisions to disclose their relevant identities, we wish to highlight how our backgrounds and experiences are relevant to the development of the STYLE framework. One author is Nigerian American; she is a Black woman and an immigrant to the US who is also a licensed clinical psychologist and an Associate Professor of psychology and public health. Another author is a Haitian American cisgender man who is an endocrinologist scientist, community leader, and a formerly incarcerated person. A third author is a White genderqueer woman; she is a public health researcher and a licensed clinical psychologist who has worked in juvenile and adult prisons. A final author is an African American, cisgender woman who holds a position as Associate Professor at a school of public health.

### The theoretical framework of STYLE across contexts

We intentionally center the implementation of STYLE within the context of a cultural humility framework, such that life-long learning, critical self-reflection, recognizing and challenging power imbalances, and promoting institutional accountability are prioritized in reaching systemic change (Tervalon and Murray-García, 1998). Structural racism is embedded in our society, encoded into laws and practices, influencing how we are raised as children, how we see and interact with the world, and how we are trained as professionals (Bailey et al., 2017; Eberhardt, 2020). Accordingly, we briefly discuss how to use STYLE to address structural, intrapersonal, and interpersonal racism. We further highlight how such work can promote equity in the legal system, and emphasize how the STYLE skills can be applied more broadly.

Figure 1 provides an accountability tool for implementing STYLE, using a cultural humility framework, as well as ways to maintain momentum in the ongoing process of developing STYLE. For each individual (and aspect of STYLE), commitment to implementation (i.e., precontemplation, contemplation, action, maintenance, termination; Prochaska, 2020) may differ. We strongly encourage the use of this monitoring form or a similar mechanism to track progress and setbacks in addition to having an accountability partner. Below we focus on how to implement each STYLE component with contextual examples focused on anti-Black racism within the legal system. Table 1 describe the STYLE application across six distinct yet overlapping contexts: advocacy and public policy, clinical care, community service, research, teaching, and training and supervision.

**Table 1:** Self-examination, talk openly, yield time, learn about racism, and evaluation of practices and policies across contexts.

	Self-examination	Talk openly about racial equity	Yield time and space	Learn about structural racism	Evaluate practices and policies
Clinical Care	Develop awareness of your biases about race and crime; Ensure your language is accurate; Engage in rupture-repair after interpersonal racism	Practice active listening techniques with patients and clients. Believe patients and clients	Consider how structural racism in the criminal legal system impacts your patients and clients	Learn common ways that the criminal legal system impacts your patients and clients through readings and conversations with patients and clients	Ensure equity in caseload, including number of patients, presenting problems, and insurance type
Community Service	Think about how you define community safety and crime in your community versus in other communities	Use your voice to dismantle systems of privilege and oppression in public spaces, particularly in discussing new legislation	Evaluate how your current community work may or may not challenge structural racism; Become involved in local efforts to reduce legal system involvement	Read about forced removal and how redlining impacted local global majority communities; Learn about local policies that could enforce or reduce structural racism	Look at local policies like drug control policy and criminal legal policies and practices (e.g., rates of policing, incarceration)
Training, mentoring, and supervision	Reflect on how you respond to trainees, mentees, and supervisees based on their identity	Discuss how policies and practices inequitably impact health; Model and co-advocate lobbying for trainees	Dedicate time during didactics and seminars to review and discuss racism, emphasizing the legal system	Improve understanding about how to practice and promote anti-racism in your mentoring work	Collect data on experiences of trainees; Examine policies associated with training through a racial bias lens
Advocacy	Think about which issues matter to you in psychology and policy; Vote and consider which elections you prioritize and why	Use your voice to prioritize policies that negatively harm members of global majority communities; Name structural racism	Include impact on structural racism as a policy evaluation criterion in policy-focused meetings; Work to ensure compensation for leadership work focused on anti-racism	Become informed on policies related to the legal system that harm members of global majority communities; Learn the history of the U.S. legal system	Monitor policy changes and data affecting policy changes, regarding native-born and immigrant global majority communities
Teaching	Assess bias in language you use when teaching; Consider whether you address structural racism in your teaching work	Use your platform as an educator to discuss structural racism	Incorporate structural racism in courses; Encourage anti-racism in coursework and teach the full history	Prioritize trainings on the legal system and on how structural racism impacts your brand of public health	Work to enforce inclusion of structural racism in curricula
Research	Examine how you describe global majority-identified communities and incorporate structural racism in your research	Measure and discuss structural racism in your research; Collaborate with other scholars who are activists	Prioritize structural racism in fora during which research is discussed; Incorporate legal involvement in your research	Learn about how the legal system impacts outcomes for communities most impacted by your research	Incorporate legal policies into research, particularly when researching racial disparities

### S: self-examination to improve awareness and address barriers to growth

In efforts toward effective anti-racism work and systemic change, self-examination and critical self-reflection are crucial in the areas of individual experiences of oppression and privilege, individually held biases, and personal reasons for inaction (Lai et al., 2014, 2016; Forscher et al., 2019; Burnett-Bowie et al., 2022). To accomplish this, individuals must raise their critical consciousness and strength and resilience to counter oppression (French et al., 2020). We must also work alongside people with lived experience (DeBlaere et al., 2019) – including those with so-called violent offenses and those occupying positions of power within the legal system (e.g., police, judges, correctional officers). Individuals must also engage in self-examination which could lead to a radical healing process that simultaneously recognizes the harms from oppression and promotes faith in justice (French et al., 2020).

#### The self-examination process

Self-examination processes largely consist of individual exercises, group workshops, and workplace trainings. Utilizing an asset-based framework where individuals with lived experience lead these trainings is encouraged (Garoutte, 2018). Individual exercises might include working with structured workbooks and can be a good starting point (e.g., Singh, 2019; Saad, 2020). Workshops are often a good next step in self-examination, as they involve self-selected collectives working toward a shared goal of anti-racism skills development. Workshops offered in person and online offer great opportunities to begin and maintain anti-racism work. BlackLivesMatter has many local chapters, as does Showing up for Racial Justice (for people racialized as White). Exercise and workshop content should facilitate participant understanding of racism as a structural or systemic problem including a historical context of policy-based oppression, should define whiteness, and should challenge common myths and norms in society (e.g., individualism, disconnect between past and present, myth of meritocracy, culture of White supremacy). Skills offered during these exercises and workshops should also include directed attention toward understanding individually held positions, systems of oppression, and consideration of additional “isms” including racism, sexism, heteronormativity, ableism, xenophobia, and classism. For further examples of skills that can promote civil courage toward anti-racism, please see Williams et al. (2023).

Self-examination and self-understanding of individual biases can impact experiences of internalized and interpersonal racism, but it is also important in addressing structural racism (Jones, 2000; Eberhardt, 2020). Through increased internal awareness of how each individual contributes to upholding structural racism and what structural racism looks like in our work (e.g., how it impacts the populations we study, teach, serve, and advocate for), we can recognize problem areas and promote or support systems-level change. Thus, STYLE promotes dedicated time toward reducing individual implicit racial bias. The most efficacious interventions to date include exposing individuals to stereotypic and counterstereotypic representations of both oppressed and privileged groups to help restructure cognitive associations (Lai et al., 2014). Limited longevity of effects from short-term implicit racial bias trainings (Lai et al., 2014; Forscher et al., 2019) and identification of barriers that could impede progress in self-examination (Howard, 2016 French et al., 2020) emphasize the need for prolonged and intentional practice of critical self-reflection, one that is ongoing and includes setbacks as individuals perpetually strive toward reaching equity. Long-term interventions and continued education through readings, self-reflection, proximity to the issue (connecting with people directly impacted by structural racism or people with lived experience), group work, and targeted trainings, could impact behavior more permanently (Williams et al., 2023).

Individuals working to impact change within a specific system might want to engage in separate, specialized self-examination work. For example, those doing work within clinical training or work associated with research or education within the legal system might consider examining their own biases about people impacted by the relevant system (e.g., incarcerated persons, legal victims) and during this work should reflect on their own experiences within the system. Reading books published by system impacted people and attending events held by local community organizations serving these groups are two ways to deepen this work.

#### Barriers to overcome during the self-examination process

Self-examination and critical self-reflection processes can allow people to situate themselves and their thoughts and actions within the larger social context of structural racism, with an emphasis on strengthening critical consciousness. Such reflection also establishes a foundation for reprogramming, realization of agency, and personal change. Still, there are several barriers to self-examination and accurate self-reflection. For example, while moving through this process, it is possible to get stuck in a state of despair or in utopian fantasies (French et al., 2020). Indeed, on the extreme ends of this spectrum, feelings of being overwhelmed can lead to disempowerment whereas having rose-tinted tunnel vision about the future can lead to a disconnect from the reality of the present. It is encouraged that individuals strive toward reconciling these two extremes by recognizing and resisting oppression while envisioning potential liberation and well-being.

**Figure 2:**
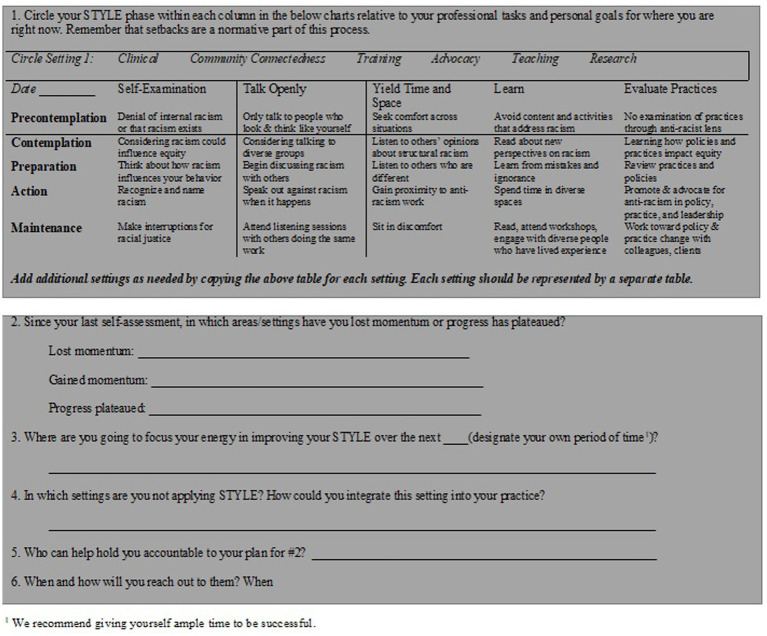
Checklist to track skills development and progress.

To help understand barriers to action and deep internal interrogation, self-examination should be accompanied by feedback from both trusted individuals and strangers alike through a 360-evaluation format (Berry et al., 2012) across contexts (see Table 1). Although self-examination is often internally exercised, identifying an accountability partner for between-person accountability is strongly advised (Goodyear, 2015). Accountability partners help ensure steady progress (see Figure 2 for ways to integrate accountability partners in monitoring plans). Accountability partners are also critical in maintaining health during a period of despair or utopian fantasies within the process of radical healing (French et al., 2020).

Another barrier that is often present for White people is an automatic tendency to internally resist and become defensive in response to internal interrogation of racial identity through responses of defensiveness (Gillespie et al., 2002). Indeed, it is important to monitor feelings of discomfort while implementing STYLE. Discomfort is a normative experience while learning and engaging in anything new and anti-racism work is no different, and therefore it is critical that each individual using STYLE or a similar framework reframe the experience of discomfort as a value that provides opportunities for learning and progress. One strategy to overcome this tendency recommended by Howard (2016) is for the affected party to consider how “the enemy is dominance itself, not White people (p. 27).”

### T: talk freely and openly about racism and the need for equitable policies and practices

In research, practice, and advocacy work, structural racism and its impact on Black communities must be directly named and addressed, and certainly not limited to observational work demonstrating race-based health inequities. Lensmire et al. (2013) explains how the discourse on privilege has largely become about White confession rather than anything resulting in meaningful outcomes; the consistent emphasis on merely “naming” racism runs that same risk. Table 1 identifies ways to talk openly about racism across contexts and provides a model of implementing STYLE for clients, students, mentees, colleagues, and more. Historically, this work has been done by Black community leaders and researchers, and there is a need for ALL who do work in psychology and related fields to communicate openly and effectively about structural racism using evidence-based approaches that ultimately support and promote policies that could promote racial equity (Davey, 2009).

#### Talking freely examples in STYLE

By freely and openly naming and measuring structural racism in teaching, training, and research, professionals in psychology and public health have the power to drive their influence beyond the academic community and to extend their reach to influence policy and practice. When teaching, naming racism might entail adoption of a race-conscious curricula that integrates research, theories, and methodologies explicitly recognizing racism as a system that harms the health of Black communities (Ford and Airhihenbuwa, 2010) or implementing existing open-source syllabi (French et al., 2019). These curricula should also include directly impacted people as the leaders and creators of these teachings and trainings when possible.

In advocacy work in government (e.g., municipal, state, federal), community (e.g., organizations increasing housing, employment, education access) and institutional (e.g., university, hospital, policing, carceral) settings, exemplary policies can be used to frame the development of new policies or argue against reauthorization of existing policies (Williams and Mohammed, 2013). Given the historically racist legacy of the U.S. legal system, there are seemingly limitless pathways and processes through which influences of structural racism are transmitted and patterned. Working collaboratively on such efforts with directly impacted people should be prioritized; thus, advocacy efforts by psychologists can begin by following the lead of grassroots organizations led by directly impacted people already doing this work (Guerrero Ramirez et al., 2022).

Examples of policies, redlining has encouraged police hyper-surveillance and overpolicing in Black communities (e.g., Such, 2018), with dire outcomes (Mitchell and Chihaya, 2021). And policies like Stop and Frisk are associated with significantly more police contact and greater use of force during police encounters for Black community members (Cooley et al., 2020). Moreover, these systems of oppression are maintained over time and codified into law.

Structural racism extends to family court contexts (e.g., Olivares, 2022) and forensic-related fields including psychology and the forensic sciences (e.g., Yim et al., 2022). Indeed, a growing body of literature based in critical race theory challenges lawyers, advocates, and educators to envision and develop strategies to use, teach, and confront the law by directly naming the role of anti-Black racism and by outlining how the legal system depends on the historic and systemic oppression of select communities to uphold the principles of White supremacy (Olivares, 2022). Black and Latine practitioners are underrepresented in forensic-related fields (Yim et al., 2022). This lack of representation in law enforcement agencies likely contributes to the lack of community trust toward the police, distrust toward forensic DNA technology, and reluctance to cooperate during criminal investigations.

### Y: yield time and space to engage more proximally in anti-racism work

People with lived experience in the legal system are often an overlooked and undervalued group and tend to be excluded from professional psychology and public health fields due to “unjust systemic issues in education and training” (Wilcox and Taylor, 2022, p. 1). Professionals working with community members who have been impacted by the legal system have an ethical imperative to get proximate to these issues. Mosley et al. (2021) co-constructed a model of Critical Consciousness of Anti-Black Racism entailing three developmental processes to be used. We recommend using these processes when yielding time to anti-racism: witnessing, processing, and acting critically against anti-Black racism. In brief, we encourage individuals to witness racism in the legal system (e.g., serving on jury duty or sitting in a courtroom for a day), process such experiences with individuals who are outside of the witnessed interaction and who are directly impacted (e.g., with colleagues or your accountability partner), and take action against anti-Black racism using your own skillsets (see suggested actions in Mosley et al., 2021 Williams et al., 2023; and Table 1).

Even among those not working directly in the legal system, it is important to recognize that many individuals we interact with will have been impacted by the legal system. Many individuals have negative experiences with police, are survivors of crime, have an incarcerated peer or family member, or know someone who is system-impacted; Black communities are frequently overrepresented in the system (Alexander, 2020) and rates of involvement may be underreported given associated stigma. Some people avoid reporting crime – especially acts involving interpersonal violence, due to understandable worries about legal ramifications and mistrust in police (Brunson and Wade, 2019). Additionally, people may have mistrust in psychologists and thus may not disclose or report traumatic events to them (Whaley, 2001). Through developing a knowledge base of the legal system and a working understanding of what system involvement could look like, individuals can ask deeper and more targeted questions, regardless of how closely one works to the legal system.

#### The processes of yielding time and gaining proximity

A great place to start advocacy work specific to improving outcomes for those in the legal system is through involvement in the increasingly supported Ban the Box movement (Weaver, 2018). “The box” references the box where people with a felony conviction are required to check on myriad applications, which limits employment, educational, and housing opportunities.

Other strategies to gain proximity to and engage in anti-racism work might include (but should not solely focus on) donating money to community-based organizations working to improve the health and well-being of lives in the Black community. Community-based organizations often encourage people to show up to events, engage virtually via petitions and virtual civic and community meetings beyond contributing financial resources. For more hands-on opportunities to engage in local anti-racism work, volunteer for events hosted by community-based organizations working with currently and formerly incarcerated people, those who have survived violence, those working with people facing homelessness, housing insecurity, and food insecurity, and those conducting trainings on structural racism and implicit bias with legal system professionals. These opportunities not only allow for direct support of the critical work of community organizations, but also provide opportunities to develop a working relationship with the organization that can become mutually beneficial as trust is built. Another advanced form of yielding time or gaining proximity to the legal system would be hosting listening events with members of directly impacted groups to inform your work; it is critical to equitably engage these individuals (Guerrero Ramirez et al., 2022). For example, research related to the legal system should be done in conjunction with those impacted by the system, and thus development of a research steering committee or community advisory board comprised of directly impacted populations to inform research questions and methods could strengthen participant receptivity and uptake of any associated interventions.

### L: learn about how to target structural racism within the legal system

To target the problem of structural racism, it is necessary to gain relevant knowledge and develop a suitable toolkit. Structural competency is important but often overlooked in training (Metzl and Hansen, 2014). It entails five principles: (1) recognizing structures that shape how people are responded to in a system, (2) developing extra-clinical language of structure, (3) rearticulating cultural presentations in structural terms, (4) understanding structural interventions, and (5) developing structural humility (see Metzl and Hansen, 2014 for a more comprehensive overview and Table 1 for recommendations across contexts).

To truly immerse oneself into anti-racist learning, one must engage and sit with people in the struggle. Researchers are encouraged to invite the wisdom and lived experiences of researchers, community members, and community organizations from Black, Indigenous, and Latine communities. It is particularly important to foster collaborative relationships and learn from people who have lived experience in the legal system as equal partners in psychology and public health research (CDC, 2011 Guerrero Ramirez et al., 2022). Through community-based participatory research (Israel et al., 1998) and equitable engagement of people with lived experience (Guerrero Ramirez et al., 2022), it is more likely that targets to address structural racism in psychology and public health research will be responsive to the community’s immediate needs and priorities.

An example of a topic that one might learn about is how members of Black communities experience a disproportionate rate of legal system involvement due to structural racism within systems of housing, employment, income, health care, education, and media. Inequities in housing (which can contribute to inequities in the legal system) in turn stem from policies such as redlining and practices such as the forced removal of Indigenous peoples from their lands in the U.S. (Hankin and Abela, 2005; see also the LANDBACK movement and Native Land). By learning about the interconnected nature of structural inequities across systems and associated health and economic costs, psychologists and other professionals can build the capacity to impact change.

Measurement of structural racism is particularly important in the fields of psychology and public health (Groos et al., 2018). Investigations of legal system inequities can identify pathways through which specific policies or patterns of policy enforcement result in disparate outcomes. It is especially important that White practitioners and professionals trust the perspectives of Black people with lived experience and recognize the power imbalances inherent in this work, from therapeutic to research settings. Key health inequities in the legal system should be recognized as relevant to psychology and other health professionals. Mental and physical health care are generally accessible to all persons in the carceral system, but the care received is often limited and outdated (Young and Badowski, 2017). People in the carceral system present with higher morbidity and greater physical and mental health care needs compared with those outside of the carceral system (Gottfried and Christopher, 2017; Nowotny et al., 2017), and there are racial inequities in these problems (Nowotny et al., 2017). Health problems (Nowotny et al., 2017) and housing insecurity (Pettit, 2012) frequently persist upon reentry into the community. In all, the interconnected systems in which people might experience structural racism contribute to health disparities that should be learned and addressed in psychology and other related professions.

### E: evaluate policies and practices that promote racism

Professionals in psychology and related fields must be knowledgeable about problems experienced – and strengths embodied – by individuals in Black-, Indigenous-, and Latine-identified communities (see Table 1 for recommendations on how to use this knowledge). In advocacy work, learning about current policy and legislative strategies to address and dismantle racism is critical (Reskin, 2012). Harmful policies including those that allow for segregation of resources and racial equity policies that create group advantage must be identified. To promote best practices in policy work, professionals in psychology and public health must consider which systems *are receiving* investment of time and resources alongside evidence about which systems *should receive* investment of time and resources. Such education is especially important to avoid repeating policy mistakes which, even if inadvertent, can and do have devastating consequences on communities for generations.

A comprehensive social justice model requires community activist perspectives and engagement from people with lived experience; advocacy across multiple levels (local, state, national, international); use of multiple methods to connect with members of the public (e.g., op-eds, podcasts, research briefs) in addition to typical academic products; and workshops in public health coursework specific to policy and implementation and dissemination of systems-level interventions (DeBlaere et al., 2019). Further, inclusion of key interconnected systems that are subject to structural racism (e.g., education, health care, housing, legal) (Bailey et al., 2017) and examination of critical explanatory (and mutable) factors is needed.

In research that is explicitly quantifying racial disparities among people in the legal system, a systems approach is necessary (McLeroy et al., 1988; Bronfenbrenner, 1992; Reskin, 2012). There is a tendency for policies and practices that impact Black communities to be more punitive and focused on social control. For example, through the examination of policies spanning school pathways to the legal system, researchers can better understand how policies must target multiple interconnected systems to effectively address structural racism, such as disproportionate involvement in the legal system. Illustratively, criminalized disciplinary approaches are more frequently implemented in predominantly Black schools (Ramey, 2015). In Black communities, there are inequitable and harsher policing practices (Hyland et al., 2015); this contributes to significantly more Black people being incarcerated *per capita* than any other racial group, despite only being 13% of the U.S. population (Puzzanchera and Hockenberry, 2020). Disproportionately harsh – even if inadvertently so – responses to citizens living in Black communities indicate where we need to target our energy. In response, researchers could examine policies specific to racial equity, and advocacy work should promote legislation such as required racial equity impact statements (Council Office of Racial Equity, 2021). This would function as an equity litmus test.

Ultimately, to do this work, researchers (including those in training) must learn: (1) what prevention and intervention techniques exist that target racism and its negative psychological and public health effects (and the effectiveness of these interventions), (2) what measures and methods are needed to prevent racism and its negative health effects, and (3) how psychology and other health professions might better examine racism and its negative health effects via implementing population-based surveys. In addition, development of policy evaluation and analytic skills should be incorporated as an elective for advanced statistical coursework requirements in doctoral-level psychology curricula (Fischer and Miller, 2017).

There are existing interventions targeting structural racism with promising effects on community-level health that researchers could evaluate independently, expand upon outcomes examined, or modify to address additional concerns specific to legal system involvement. For example, place-based, multisector, equity-oriented initiatives like Purpose Built Communities and Promise Neighborhoods encourage large-scale interventions that focus on changes to housing to reduce structural inequities (Bailey et al., 2017). Interventions focused on educating and employing people (especially Black people) are particularly effective at reducing recidivism and are a cost-effective alternative to incarceration (Visher et al., 2008; Davis et al., 2013) but more evaluation work is needed.

### Evaluation of STYLE

Immediate next steps for strengthening and promoting the refinement of the STYLE framework are underway, though we encourage others to also test and improve upon the STYLE framework we propose. There is a need to examine the data on how effectively STYLE can translate to behavior change and ultimately contribute to change that promotes health equity among the populations with whom we work. Evaluative work of STYLE will include data collection from professionals about their perceptions of STYLE, successes and difficulties in implementing the STYLE framework, and behavior and cognitive changes over time. Data should also be collected directly from people with lived experience through focus groups and listening sessions to iteratively refine content that needs updating and eventually to measure outcomes from a subset of people in the legal system.

#### Areas for consideration in testing STYLE

Though we are promoting implementation and evaluation of STYLE, it was developed as a foundational yet dynamic framework, partly because we hope it can be used to promote anti-racism work while considering intersectional identifiers and using unique skillsets. But we recognize both intersectional identifiers and required skillsets may change over time. Further, we have not tested STYLE with a wider audience of psychologists, namely those working outside of medical or forensic/legal or across advocacy and public policy, clinical care, community service, research, teaching, and training and supervision contexts. Finally, the STYLE framework could also be limited in uptake given that its implementation requires ongoing and emotionally challenging work. Thus, it is likely that motivation to engage in the process must be high for individuals to begin and sustain their practice. While it is possible to incorporate motivational interviewing practices into STYLE implementation, no evidenced-based methods have been explored yet.

## Conclusion

Structural racism is a pernicious force in the juvenile and criminal legal systems and the broader legal system, with widespread effects on public health. The criminal legal system is interconnected with a myriad of other systems that also propagate structural racism. Education about and intentional alteration of the STYLE framework could be prioritized to effectively combat racism and promote anti-racism practices in multiple other systems. The fields of psychology and public health are perfectly poised to respond to health inequities through clinical care, education, advocacy, intervention, and research that addresses structural racism.

Structural racism is already integrated into select models of equity, but factors associated with health inequities are often overlooked within the criminal legal system (Bailey et al., 2017). Further, less is known about the actual implementation and uptake of structural racism changes within these models of equity. Our proposed STYLE framework offers a set of practices and progress tracking guide to promote anti-racism among public health professionals, particularly considering structural racism in the criminal legal system. Engage in *Self-examination* to encourage more individual and organizational growth and risk in anti-racism work. *Talk* freely and openly about promoting equitable policies and practices targeting the problem of racism in the U.S. Recognize that all spaces are impacted by structural racism and thus, *Yield* time and space to anti-racism work. *Learn* through increased proximity about structures, policies, practices/norms, and values in the spaces in which we work as professionals. *Evaluate* existing policies and practices using the equity litmus test (similar to a fiscal impact analysis but for equity).

We intentionally centered our paper around Black adults in the criminal legal system given the overrepresentation of this population within this system; however, Black youth and adults and youth from other minoritized racial and ethnic groups are also harmed by the legal system. Specifically, inequities extend to Black children in particular who are twice as likely to be sentenced to out-of-school suspension compared with White children (Camacho and Krezmien, 2020), and 32% of youth placed in secure facilities after being adjudicated delinquent by the juvenile courts identify as Black; a clear overrepresentation given that they account for only 18% of the population (Puzzanchera and Hockenberry, 2020; U.S. Census Bureau, 2020). Moreover, many other systems impacted by structural racism and oppression can be considered when implementing STYLE (Williams and Mohammed, 2013; Bailey et al., 2017), as can other intersectional identities like gender identity, class, disability, and sexual orientation (Crenshaw, 1989; Bauer, 2014).

Some challenges experienced by our group have been ensuring STYLE meets the needs of psychologists across multiple roles and contexts. While STYLE was developed with the legal system in mind, the aim of the framework is application to other systems (e.g., education, child welfare and social services, medical). Our group does not include psychologists who represent all roles and contexts, and in fact includes collaborators with training in public health. During iterative refinement of the STYLE components, the different needs and foci of these two overlapping but distinct fields clashed at times. We also anticipate possible difficulties balancing perspectives and fostering open discussion about whose voices should be prioritized in situations of disagreement, and who should be leading this work. Our collective agreement is to prioritize the voices and perspectives of people with lived experience and to support them as leaders of this work when possible.

To effectively upend racism and practice anti-racism in psychology and related fields like public health, there is an urgent need to focus on educating the next generation of professionals – and helping them to intentionally alter their STYLE – through modeling and dedicated practice.

## Data availability statement

The original contributions presented in the study are included in the article/supplementary material, further inquiries can be directed to the corresponding author.

## Author contributions

RF developed the initial STYLE framework. All authors contributed to the writing of the current paper and conceptualization of study tables and figures.

## Conflict of interest

The authors declare that the research was conducted in the absence of any commercial or financial relationships that could be construed as a potential conflict of interest.

## Publisher’s note

All claims expressed in this article are solely those of the authors and do not necessarily represent those of their affiliated organizations, or those of the publisher, the editors and the reviewers. Any product that may be evaluated in this article, or claim that may be made by its manufacturer, is not guaranteed or endorsed by the publisher.
